# Managing the toxicities of the FEC-100 followed by docetaxel regimen: The price of success

**DOI:** 10.3747/co.v15i2.263

**Published:** 2008-04

**Authors:** Kara Laing

**Affiliations:** Medical Oncologist Cancer Care Program Associate Professor Memorial University of NL

## CASE STUDY

A 52-year-old postmenopausal woman presented with an abnormal screening mammogram showing a suspicious area in the upper outer quadrant of her left breast. Core biopsy confirmed an infiltrating ductal carcinoma. She elected to have a mastectomy, and pathology revealed a 2.6-cm moderately differentiated ductal carcinoma with 3 of 16 lymph nodes positive. Hormone receptors and human epidermal growth factor (her2) were negative by immunohistochemistry. Past medical history and review of systems were unremarkable. Staging workup was negative, and baseline ejection fraction was 60% on multiple-gated acquisition scan.

This patient was offered fluorouracil, epirubicin, and cyclophosphamide followed by docetaxel adjuvant chemotherapy. She was quite concerned about the potential toxicities of chemotherapy and hoped to continue to work part-time managing her own business during treatment.

How can the potential toxicities of this adjuvant chemotherapy regimen be optimally managed?

## DISCUSSION

Many advances have been made in adjuvant therapy for early-stage breast cancer, contributing to an overall decline in mortality. One major advance has been the addition of taxanes, either concurrently with or sequentially to anthracycline-based adjuvant chemotherapy. The most recent Early Breast Cancer Trialists’ Collaborative Group (2005–2006) overview demonstrated a 5.1% improvement in overall survival at 10 years for taxane-containing regimens as compared with anthracycline regimens^a^.

The pacs 01 trial compared 6 cycles of adjuvant fec-100 chemotherapy (5-fluorouracil 500 mg/m^2^, epirubicin 100 mg/m^2^, cyclophosphamide 500 mg/m^2^) given intravenously every 3 weeks with 3 cycles of fec-100 followed by 3 cycles of docetaxel 100 mg/m^2^ given every 3 weeks (fec-d) in node-positive breast cancer patients. The sequential taxane containing arm was superior, with an improvement seen in disease-free survival [hazard ratio (hr): 0.82; 95% confidence interval (ci): 0.69 to 0.99; *p* = 0.034] and overall survival (hr: 0.73; 95% ci: 0.56 to 0.94) after 5 years of follow-up^1^.

In terms of toxicity, more nausea and vomiting, more grades 3 and 4 neutropenia, and greater use of granulocyte colony–stimulating factor was seen in the fec-100 arm. More febrile neutropenia and stomatitis were seen in the fec-d arm. Fewer cardiac events and acute leukemias occurred in the fec-d group because of the lower cumulative anthracycline dose.

The fec-d regimen was quickly adopted by Canadian medical oncologists. Many were already using fec-100 as a standard regimen for node-positive and high-risk node-negative patients. Single-agent docetaxel was a standard regimen for metastatic disease, although dose reductions to 70–80 mg/m^2^ were commonly used in that patient group. However, in the adjuvant setting, docetaxel 100 mg/m^2^ is used, and many oncologists reported seeing increased patient toxicity, especially fatigue, myalgias, and febrile neutropenia.

One of the key factors in toxicity management is patient education. At the initial oncology consultation, patients are given a vast amount of information regarding their diagnosis, prognosis, and treatment options. Verbal and written information on potential side effects of chemotherapy and their management are provided by physicians, nurses, and pharmacists. Patients often do not absorb all this important information on first delivery, and they often need it to be repeated. Before the docetaxel portion of the fec-d regimen begins, my institution reviews the toxicities of docetaxel, highlighting the differences as compared with fec-100. The goals of adjuvant treatment are also reviewed. Patients receiving their chemotherapy in our regional cancer centres are often seen in our tele-oncology clinics.

Fatigue is a common side effect of chemotherapy, and this fatigue can be both distressing and debilitating. It is often cumulative, taking longer to improve with each subsequent chemotherapy cycle. With docetaxel, onset of fatigue comes earlier—usually 3–5 days postdrug. Patients have to be cautioned that they will likely be *more* fatigued, that the symptoms will start earlier, and that they may take longer to recover.

Myalgias and arthralgias are a common toxicity of docetaxel, and these side effects may be severe. Patients have to be counselled and provided with appropriate analgesia. A longer duration of steroids may also be helpful.

Febrile neutropenia is common with docetaxelcontaining regimens. Primary prophylaxis is routinely used in anthracycline–taxane combination regimens such as tac (docetaxel, doxorubicin and, cyclophosphamide). It may also be considered when using adjuvant docetaxel 100 mg/m^2^, especially for patients at higher risk of neutropenia and its complications.

In striving to better predict who really needs adjuvant chemotherapy and to develop better predictors of chemotherapy response, attempts must be made to maximize benefit and minimize toxicity with available chemotherapy regimens. The fec-d regimen is clearly one that offers benefits in diseasefree and overall survival to patients, and with care, its additional toxicities can be managed. The best approach involves a multidisciplinary team effort, resulting in a well-informed and supported patient. And in the end, improvement in the patient’s outcome is worth it.

## Footnotes

a Peto R, for the Early Breast Cancer Trialists’ Collaborative Group. The worldwide overview: new results for systemic adjuvant therapies. Presentation at the 30th Annual San Antonia Breast Cancer Symposium; December 13–16, 2007; San Antonio, TX, U.S.A.
